# Relationships of actual and expected levels of income with GP job satisfaction: repeated cross-sectional study

**DOI:** 10.3399/BJGP.2025.0683

**Published:** 2026-08-25

**Authors:** Benjamin Walker, Michael Anderson, Ravi Ganepola, Joseph Hutchinson, Jonathan Gibson, Sean Urwin, Katherine Checkland, Matt Sutton

**Affiliations:** 1 Division of Population Health, Health Services Research and Primary Care, University of Manchester, Manchester, UK; 2 The Old Church Surgery, The Silverthorn Centre, London, UK

**Keywords:** general practice, income, job satisfaction, primary health care, reimbursement, workforce planning

## Abstract

**Background:**

Income is known to be a key driver of job satisfaction, especially when compared with expected income. The pertinence of this for GPs is not fully understood.

**Aim:**

To examine whether GP job satisfaction and intentions to quit are affected by actual and expected levels of income.

**Design & setting:**

Repeated cross-sectional study involving GPs in England. The authors analysed responses to four waves of the GP Worklife Survey (a survey of GPs in England), between 2015 and 2021.

**Method:**

Linear regression models were used to examine the associations of actual income with 10 domains of job satisfaction and three aspects of intentions to quit, adjusted for potential confounders; responders’ self-reported expected incomes were then added to these models. These analyses were repeated, stratifying by contract status and age.

**Results:**

Higher actual income had a statistically significant association with increased satisfaction across most job satisfaction domains except for ‘Amount of variety in your job’, ‘Your colleagues and fellow workers’, and ‘Physical working conditions’. There was little evidence of a relationship between actual income and intentions to quit. Higher expected income, conditional on actual income, had a statistically significant association with reduced job satisfaction across all domains and increased intentions to quit. The relationship between expected income and intentions to work abroad in the next 5 years was stronger for GPs aged <40 years, and the relationship between actual income and job satisfaction was stronger for GP partners and for GPs aged >50 years.

**Conclusion:**

Higher incomes were associated with higher job satisfaction, but lower than expected levels of income were important sources of dissatisfaction and intentions to quit.

## How this fits in

Recruitment and retention of GPs remains an issue for general practice in England. This work examined the relationship between actual income and income expectations with job satisfaction and intentions to quit. Higher actual income was found to be associated with job satisfaction, but there was little evidence to show that it was associated with intentions to quit. A key and novel finding was that higher income expectations were associated with both lower job satisfaction and higher intentions to quit. The findings suggest that policies that help GPs build accurate income expectations, such as increased transparency for incomes for different GP job roles, may be a means of addressing job dissatisfaction caused by discrepancies between expected and actual income for GPs.

## Introduction

Recruitment and retention of GPs continues to be a major challenge in many countries. As of April 2024, there were 27 606 fully qualified full-time equivalent (FTEs) GPs in England, which is 6% lower than in September 2015.^1,2^ Demand has continued to increase during this period: the number of patients per FTE GP in England increased by 18% between September 2015 and April 2024 (from 1938 to 2294 patients per FTE GP).^3^ Patient demand has also become increasingly complex, with higher levels of multimorbidity leading to increasingly complicated presentations and greater disease burdens in practice populations.^4,5^ A recent study of UK-trained doctors working in the UK and abroad, commissioned by the General Medical Council, found that GPs were the most likely to experience ‘deep discontent’ practising medicine in the UK relative to other types of doctors, and that more than one-fifth of GPs were very likely or fairly likely to move abroad in the next 12 months.^6^

Job satisfaction is known to be a key determinant of the recruitment and retention of GPs,^7–9^ and understanding the extent to which different factors affect GP job satisfaction is key to improving the optimal supply of GPs to meet patient demand. Beyond the recruitment and retention implications, GP job satisfaction is also important because it predicts quality of care, including continuity of care, accessibility, and communication.^10,11^ There are several factors that may influence job satisfaction in the healthcare sector, including working conditions, organisational environment, job stress, role responsibilities, and perception.^12–15^ For GPs, notable attention has been paid to income as a determinant of job satisfaction: the Commonwealth Fund’s 2022 International Health Policy Survey of Primary Care Physicians across 10 countries, for example, showed that only 23% of UK GPs were satisfied with their income, which was the lowest rating across all countries, except for Australia at 17%.^16^ Despite the importance of the topic, there is limited evidence on the link between income and job satisfaction for GPs. A previous study of GP survey data from England between 2004 and 2008 found a positive relationship between income and job satisfaction.^17^ A systematic review of factors influencing GP recruitment and retention found that low income was associated with reduced job satisfaction, but there was little evidence to support the use of lump-sum payments to improve GP retention.^7^

Alongside actual income, there is pre-existing literature suggesting that relative income and income expectations also matter to individuals.^18–20^ Economic theory — namely the Easterlin paradox — notes that, as a country’s average income increases, average happiness does not necessarily increase, suggesting that relative income matters more for happiness than absolute income.^21^ Research in both a cross-sectoral sample of workers and a sample of the general population has highlighted the importance of comparisons, with income expectations being a significant driver of mental health, and satisfaction with level of income.^19,22^ These expectations may involve individuals making comparisons with peers who have different levels of experience, work abroad, or work in other specialties or sectors.^21^ Several articles have demonstrated that the choice of reference group is important and suggest more direct measurement of income expectations, captured through self-perceived relative income, be used.^23–25^ As choice of reference group impacts how individuals perceive their relative income, a direct measure of expected income works better because it allows participants to conceptualise themselves as their comparator group. If a discrepancy between expected and actual income is more important, this may explain the evidence of a weak effect of actual income.

Shortfalls in what GPs actually receive and what they consider to be an adequate level of income for their job may be a key driver of reduced job satisfaction and, therefore, also recruitment and retention. This study examined the relationships of actual and expected incomes with GP job satisfaction and intentions to quit. Although the relationship between actual GP income and job satisfaction was examined using data from 2008 in a previous study,^17^ GP incomes and working conditions have changed considerably since then. To the authors’ knowledge, no study has analysed the relationship between expected GP income and job satisfaction.

## Method

### Study cohort

Data were used from four waves of the GP Worklife Survey (GPWLS) in England conducted in 2015, 2017, 2019, and 2021. The GPWLS is a national survey conducted every 2–3 years. Alongside several other measures of the working lives of GPs, the GPWLS includes information on hours worked per week, contract status (partner versus salaried or locum), and personal characteristics (gender, ethnicity, age, years from qualification, and whether the GP is a senior partner). Supplementary Table S1 contains information on the survey periods for each survey wave.

The 2015, 2017, and 2019 survey waves were postal surveys, with questionnaires sent to a random sample of 4000–5000 GPs; the waves included an option to complete the survey online. The 2021 survey was conducted primarily using an online survey with invitation emails sent out to practices by their local clinical research network; in addition to the online survey, a small number of GPs received a paper survey.

The GPWLS includes 10 questions related to job satisfaction (to be answered using a numerical scale: 1 = extremely dissatisfied, 7 = extremely satisfied), three questions related to intentions to quit direct patient care (scale capturing likelihood of quitting: 1 = none, 5 = high likelihood), and two questions related to actual and expected income from GP work. Actual income received was reported using £20 000 intervals spanning from <£50 000 to ≥£170 000. Expected income was measured as a continuous variable using the following question: *‘Considering your experience and career stage, what do you consider to be an adequate income from your job as a GP, after expenses but before taxes?’*. Examples of items that can be expensed include staff, premises maintenance, IT equipment, and medical equipment. The wordings of other questions and possible responses used in this study are provided in Supplementary Table S2. These questions were included consistently across the four survey waves.

### Survey weighting

The profile of responders to the GPWLS over-represents the 50–59-year-old age group and GP partners (see Supplementary Table S3). To account for this, the authors used survey weights generated using GP workforce headcount data from NHS England for the corresponding midpoint of each survey wave.^1^ These headcounts were stratified by combinations of age, sex, and contract type (that is, GP partners, salaried GPs, and locum GPs), and weights were generated to provide the same distribution in the survey data.

### Analysis

Descriptive statistics for survey responder characteristics and responses were produced for each survey wave using mean and standard deviations. To assess differences across survey waves, analysis of variance tests were conducted.

Linear regression was used to examine the relationship between actual income from GP work and the 10 domains of job satisfaction, as well as the three domains of intentions to quit. Linear regression was also used to examine the relationship between expected income from GP work and the 10 domains of job satisfaction, as well as the three domains of intentions to quit, conditional on actual income received from GP work. This is equivalent to analysing the gap between actual and expected income from GP work, and benefits from not involving any imputation to address the banded information on actual income. These linear regression models were repeated to examine intentions to quit direct patient care, rather than domains of job satisfaction, and were run with and without survey weights to ascertain whether this changed the results.

Data from all four waves were pooled to increase the sample size, and indicators for each survey wave were included in the regression models to account for changes over time, including differences in methods of data collection and inflation. Responder characteristics (age, gender, ethnicity, contract status, and marital status) were also included as covariates. Age squared was included to allow for non-linear associations between age and the outcomes, and to improve model fit. Standard errors were used that were robust to heteroskedasticity. Binary variables were used for gender (1 = female), ethnicity (1 = White), and contract status (1 = GP partner). Interval variables with 10-year bands were used for age and years qualified. Marital status (single, married/cohabiting, separated/divorced, and other) was treated as a categorical variable. Stratified analyses were also conducted focusing on subgroups of GPs defined by age (<40 years, 40–50 years, and >50 years), and contract status (partner or non-partner GP). GPs were not linked to practices over time as the practice code variable was recorded inconsistently.

### Missing data

In total, 33% (*n* = 2772/8396) of observations had some missing data. Supplementary Table S3 shows the level of missingness in each variable. The variables with the highest level of missing data were expected income (*n* = 1356 [16% of total observations]) and actual income (*n* = 354 [4% of total observations]). The authors chose not to perform multiple imputation for missing values of variables, since GPs who declined to report their actual or expected income may have differed systematically from those who did, meaning the missing values may not have been missing at random.

### Patient and public involvement

No patients were involved in the design of the study as it focused on the perspectives of GPs. Consultation with groups of GPs was undertaken during the design and piloting of the survey prior to its distribution to the intended responders.

## Results

### Descriptive statistics

There were only small differences in most responder characteristics between survey waves (Table 1). The exception was a lower proportion of GP partners responding to the 2021 survey wave than the earlier waves (69.7% in the 2021 wave compared with >80% across all other waves).

**Table 1.** Descriptive statistics of variables by survey wave

Variable	2015 survey	2017 survey	2019 survey	2021 survey	ANOVA test of differences between surveys, F-stat (*P*>F)	
**Drivers of job satisfaction,** ^**a**^ **mean (SD)**						
Physical working conditions	5.19 (1.45)	5.18 (1.49)	5.18 (1.46)	5.12 (1.47)	1.07 (0.3621)	
Freedom to choose your own method of working	4.52 (1.43)	4.75 (1.45)	4.75 (1.46)	4.78 (1.46)	16.06 (0.0000)	
Your colleagues and fellow workers	5.69 (1.21)	5.72 (1.23)	5.76 (1.27)	5.73 (1.23)	1.01 (0.3893)	
Recognition you get for good work	4.22 (1.57)	4.33 (1.60)	4.54 (1.59)	4.37 (1.64)	12.75 (0.0000)	
Amount of responsibility you are given	4.84 (1.59)	4.77 (1.68)	4.94 (1.67)	4.94 (1.56)	5.96 (0.0005)	
Your remuneration	4.19 (1.60)	4.30 (1.66)	4.60 (1.71)	4.66 (1.61)	41.70 (0.0000)	
Opportunity to use your abilities	4.88 (1.40)	4.95 (1.42)	5.18 (1.40)	5.05 (1.41)	15.21 (0.0000)	
Your hours of work	3.48 (1.74)	3.61 (1.77)	3.74 (1.78)	3.70 (1.82)	9.58 (0.0000)	
Amount of variety in your job	5.21 (1.36)	5.18 (1.39)	5.28 (1.41)	5.06 (1.41)	8.03 (0.0000)	
Taking everything into consideration, how do you feel about your job? (Overall job satisfaction)	4.09 (1.49)	4.26 (1.52)	4.42 (1.53)	4.30 (1.54)	16.24 (0.0000)	
**Intentions to quit,** ^**b**^ **mean (SD)**						
Continue with medical work but outside the UK within 5 years?	1.69 (1.13)	1.63 (1.05)	1.56 (1.01)	1.59 (1.05)	5.93 (0.0005)	
Leave direct patient care within 5 years?	2.95 (1.61)	3.02 (1.59)	3.05 (1.62)	2.70 (1.55)	21.00 (0.0000)	
Leave medical work entirely within 5 years?	2.79 (1.62)	2.85 (1.62)	2.89 (1.65)	2.48 (1.54)	27.79 (0.0000)	
**GP characteristics, mean (SD)**						
Expected income from GP work, £	115 319 (37 834)	116 423 (41 068)	121 279 (44 918)	106 948 (43 042)	32.36 (0.0000)	
Hours worked per week	42.26 (13.79)	41.15 (13.62)	40.97 (18.29)	38.53 (13.79)	27.04 (0.0000)	
Age, years	49.66 (8.44)	50.23 (8.31)	50.80 (7.99)	46.81 (9.20)	87.91 (0.0000)	
**Marital status,%**						
Single	3.0	3.7	4.2	4.9	—	
Married/cohabitating	90.5	90.2	90.0	90.0	—	
Separated/divorced	5.9	5.6	5.2	4.2	—	
Other	0.6	0.5	0.7	0.9	—	
**Actual income from GP work, %^c^**						
<£50 000	10.6	11.4	10.3	14.8	—	
£50 000–£69 999	14.4	14.2	14.5	20.1	—	
£70 000–£89 999	20.9	19.2	17.7	17.7	—	
£90 000–£109 999	25.3	25.7	22.7	18.5	—	
£110 000–£129 999	16.0	14.6	15.8	13.8	—	
£130 000–£149 999	6.7	7.9	8.6	7.5	—	
£150 000–£169 999	3.1	3.6	4.3	3.5	—	
≥£170 000	2.9	3.4	6.2	4.1	—	
**GP characteristics**						
Gender (female), %	46.8	48.0	48.5	58.4	—	
Ethnicity (White), %	82.5	82.0	82.8	79.3	—	
Employment (partner), %	87.7	87.0	82.0	69.7	—	
Total number of observations, *n*	2615	2289	1390	2102	—	

^a^1 = extremely dissatisfied, 7 = extremely satisfied. ^b^1 = none, 5 = high. ^c^Reported income from GP work is a categorical variable with the following values: 1 = <£50 000; 2 = £50 000–£69 999; 3 = £70 000–£89 999; 4 = £90 000–£109 999; 5 = £110 000–£129 999; 6 = £130 000–£149 999; 7 = £150 000–£169 999; 8 = ≥£170 000. ANOVA = analysis of variance. SD = standard deviation.

Small but significant differences were found between waves for all job satisfaction domains, except ‘Physical working conditions’ and ‘Your colleagues and fellow workers’. Significant differences were also found between waves for all three domains of intentions to quit, expected income, hours worked per week, gender, ethnicity, and age. Trends in the outcome measures over time can be observed in Table 1. For job satisfaction domains, a steady increase in satisfaction was observed from 2015 to 2021 for ‘Freedom to choose your own method of working’ and ‘Your remuneration’. For ‘Recognition you get for good work’, ‘Opportunity to use your abilities’, ‘Your hours of work’, and ‘Taking everything into consideration, how do you feel about your job?’, increases in mean satisfaction were found from 2015 to 2019, and a small decline was observed from 2019 to 2021. For ‘Amount of responsibility you are given’, there was a slight decline in mean satisfaction from 2015 to 2017, but an increase from 2017 to 2019 and no change from 2019 to 2021. For ‘Amount of variety in your job’, there was a slight decrease from 2015 to 2017, an increase from 2017 to 2019, and then a decrease from 2019 to 2021.

Regarding trends in intentions to quit, there was a small decline in intentions to work abroad from 2015 to 2019, before a slight increase in 2021. For both ‘Leaving direct patient care within 5 years?’ and ‘Leaving medical work entirely within 5 years?’, there was a steady increase from 2015 to 2019, before a decline in 2021 (Table 1).

The modal band of expected income for the sample was £90 000–£109 999 (see Supplementary Figure S1). Figure 1 displays the average expected income by category of actual income. For all income bands up to £130 000–£149 999, mean expected income was higher than the upper bound of the actual income category. For the highest two actual income categories, average expected incomes were lower than the upper bound of the actual income category; in other words, relatively low earners expected to earn more and relatively high earners expected to earn less.

**Figure 1.**
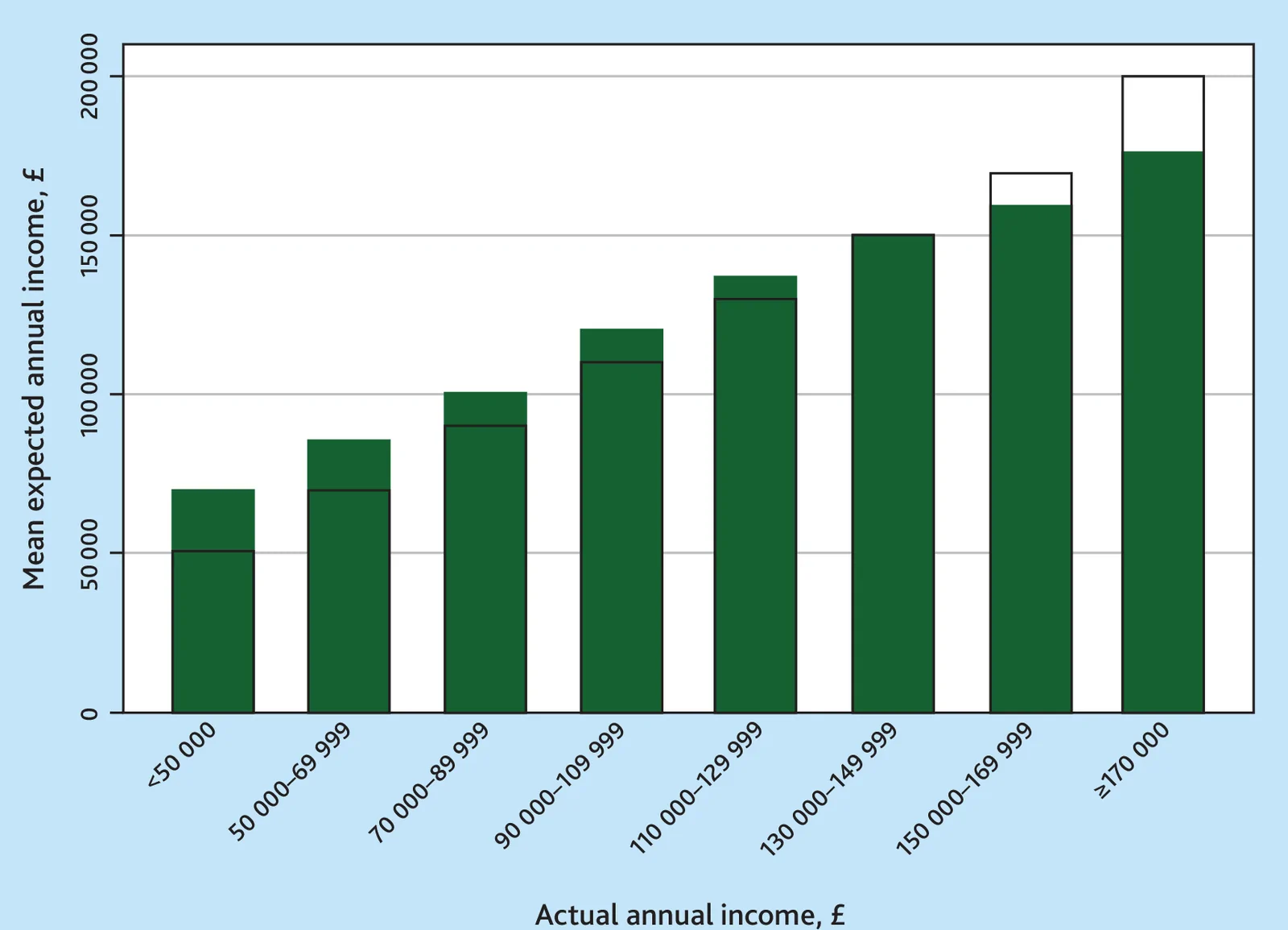
Mean expected income by actual income bracket. Green represents the mean expected income of those in an actual income band. Black outlines show highest actual income within an income band, except for ≥£170 000, for which a round figure of £200 000 has been inserted for visual clarity in place of an actual maximum*.*

### Main results

Figure 2 shows the relationship between actual income and the 10 measures of job satisfaction. Higher actual income was associated with significantly increased satisfaction across most domains of job satisfaction except for ‘Amount of variety in your job’ and ‘Your colleagues and fellow workers’. Compared with a baseline income of <£50 000 per year, overall job satisfaction increased significantly once actual income reached >£90 000. Full regression results are provided in Supplementary Table S4.

**Figure 2.**
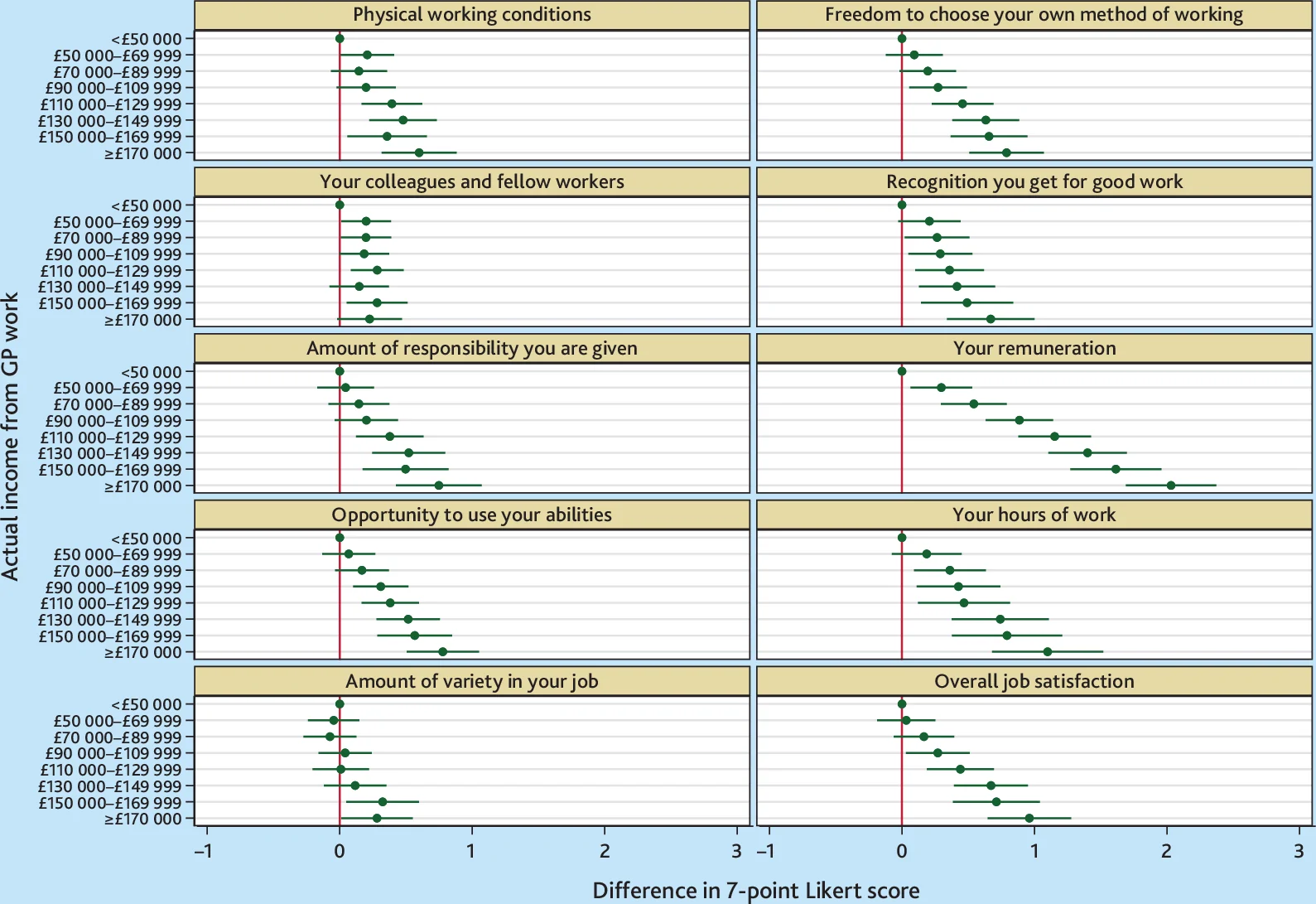
Estimated difference in job satisfaction across categories of actual income. Job satisfaction scored 1–7 (1 = extremely dissatisfied, 7 = extremely satisfied). Results are estimated using linear regression with robust standard errors. Exposure is GPs’ actual income in £20 000 bands. Adjustment was made for individual characteristics including partner contract, gender, marital status, ethnicity, age, hours worked, and year. The coefficient presented is from the pooled analysis involving all survey waves.

Figure 3 shows the relationship between actual income and three measures of intentions to quit. Increasing actual income was associated with a trend towards reduced intentions to leave direct patient care and leave medical work entirely within the next 5 years, although no effect of any individual income category was significantly different from the lowest income category. Compared with the lowest category, incomes of £50 000–£69 999, £70 000–£89 999, and £90 000–£109 000 were associated with increased intentions to continue medical work abroad within the next 5 years: 0.237 (95% confidence interval [CI] = 0.068 to 0.406), 0.251 (95% CI = 0.094 to 0.407), and 0.192 (95% CI = 0.034 to 0.350), respectively. However, the relationship between higher actual income and intentions to continue to work abroad in the next 5 years were not consistent. Full regression results are included in Supplementary Table S4.

**Figure 3.**
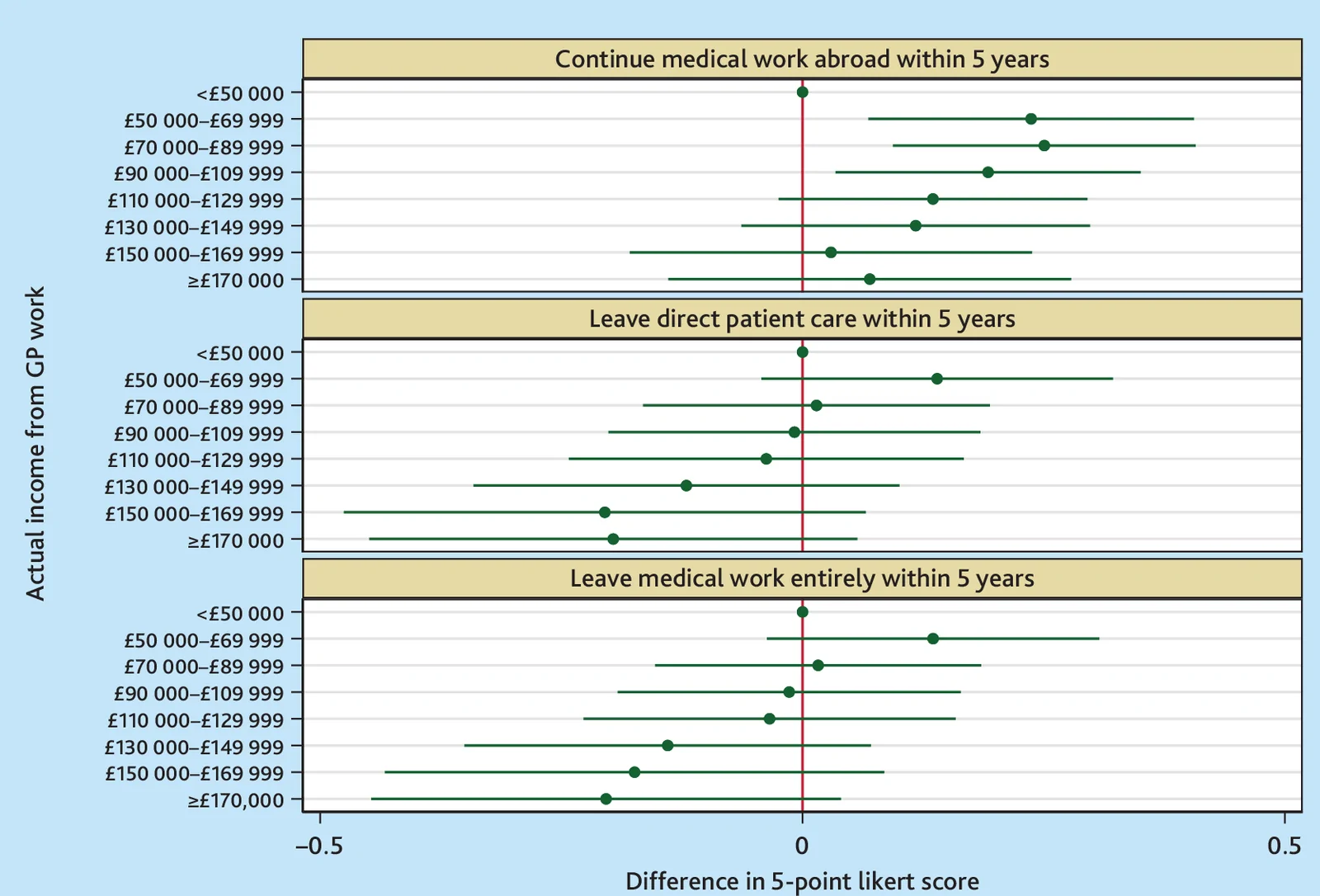
Estimated differences in intentions to quit across categories of actual income. Intentions to quit score 1–5 (1 = none, 5 = high likelihood). Results are estimated using linear regression with robust standard errors. Exposure is GPs’ actual income in £20 000 bands. Adjustment was made for individual characteristics, including partner contracts, gender, marital status, ethnicity, age, hours worked, and year. The coefficient presented is from the pooled analysis involving all survey waves.

Figure 4 shows that a £10 000 higher expected income, conditional on actual income, was associated with reduced overall GP job satisfaction (0.092, 95% CI = 0.076 to 0.108) and reduced job satisfaction on nine specific domains. The largest reductions in job satisfaction were with remuneration (0.193, 95% CI = 0.177 to 0.210) and GPs’ opportunity to use their abilities (0.086, 95% CI = 0.067 to 0.102). Full regression results are shown in Supplementary Table S5.

**Figure 4.**
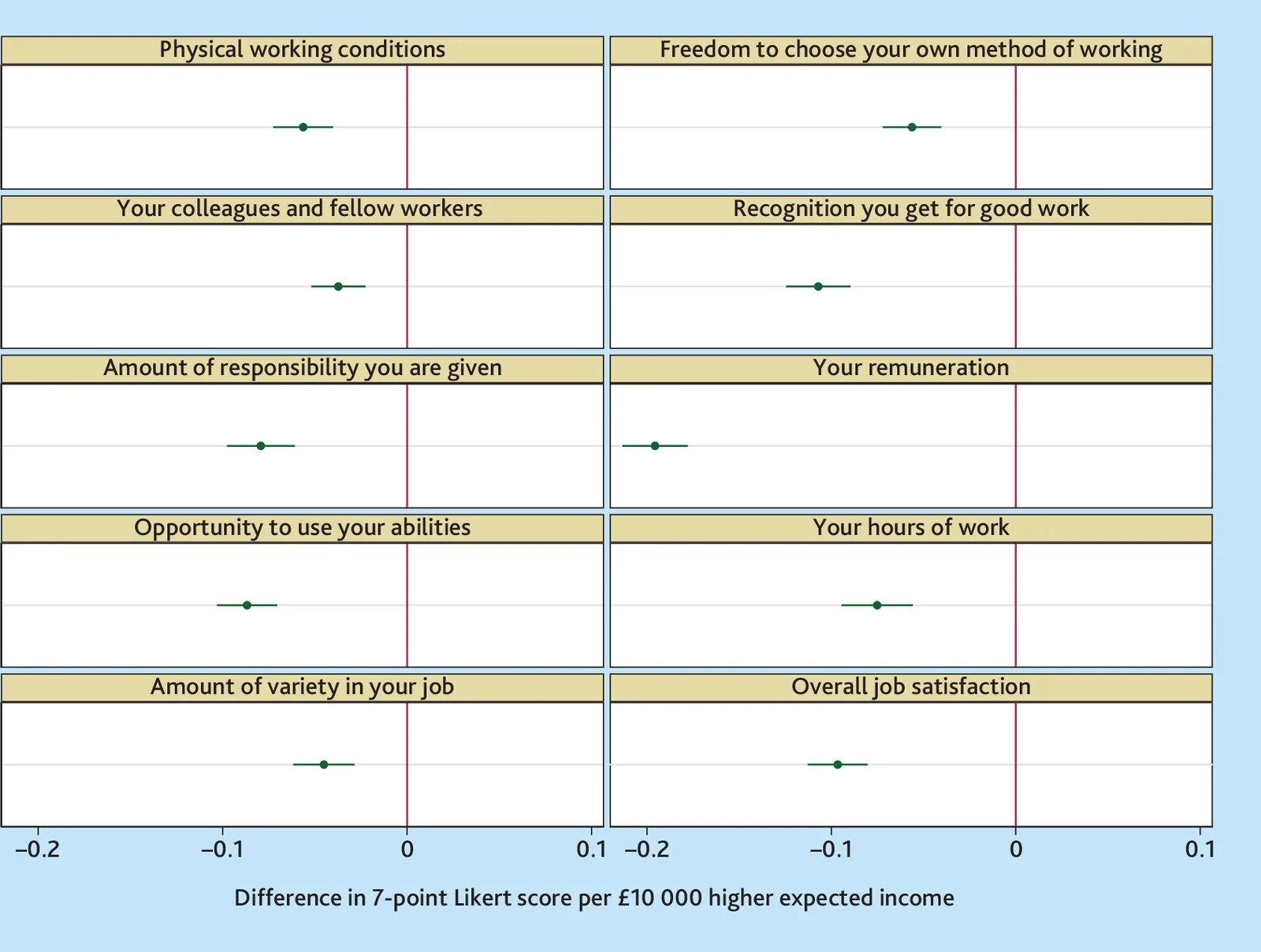
Estimated effects of expected income (conditional on actual income) on job satisfaction. Job satisfaction scored 1–7 (1 = extremely dissatisfied, 7 = extremely satisfied). Results are estimated using linear regression with robust standard errors. Exposure is GPs’ expected income/£10 000. Adjustment was made for individual characteristics, including partner contract, gender, marital status, ethnicity, age, hours worked, actual income (categorised), and year. The coefficient presented is from the pooled analysis involving all survey waves. As actual income has been adjusted for, higher values of expected income can be viewed to represent a larger gap between expected income and actual income.

Figure 5 shows that a £10 000 higher expected income, conditional on actual income, was associated with all three indicators of intentions to quit, including intentions to continue medical work abroad (0.052, 95% CI = 0.039 to 0.064), intention to leave direct patient care (0.040, 95% CI = 0.026 to 0.054), and intention to leave medical work entirely (0.033, 95% CI = 0.019 to 0.046) within 5 years. Full regression results are shown in Supplementary Table S5.

**Figure 5.**
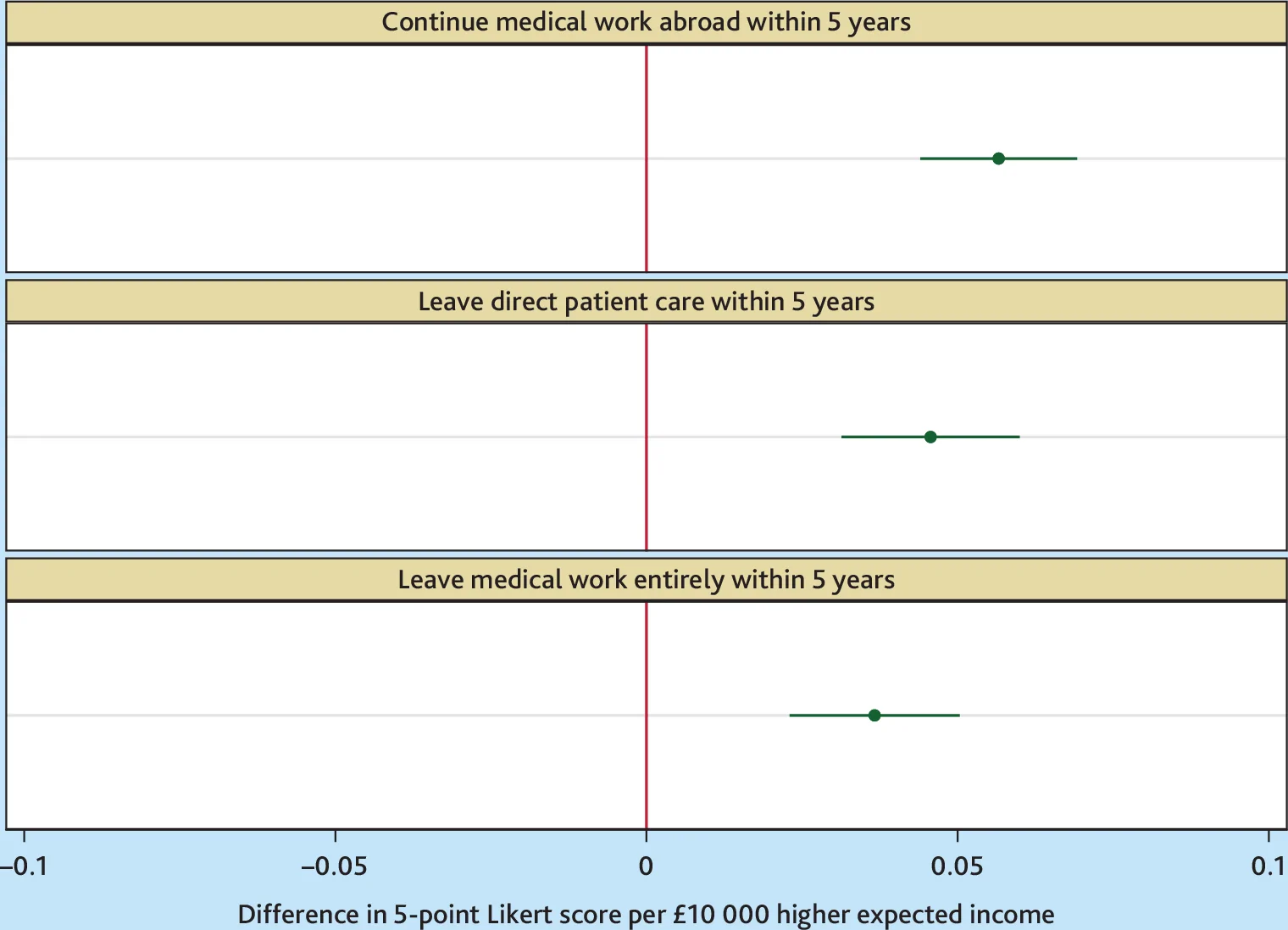
Estimated effects of expected income (conditional on actual income) on intentions to quit. Intentions to quit scored 1–5 (1 = none, 5 = high likelihood). Results are estimated using linear regression with robust standard errors. Exposure is GPs’ expected income/£10 000. Adjustment was made for individual characteristics, including partner contracts, gender, marital status, ethnicity, age, hours worked, actual income (categorised), and year. The coefficient presented is from the pooled analysis involving all survey waves. As actual income has been adjusted for, higher values of expected income can be viewed to represent a larger gap between expected income and actual income.

### Supplementary analyses

Removing survey weights did not change the results, with similar results for actual and expected income as in the weighted analysis (see Supplementary Figures S2–S5).

Focusing the analysis on non-partner GPs, there appeared to be no consistent relationship between actual income and job satisfaction and intentions to quit (see Supplementary Figures S6 and S7); however, higher expected income, conditional on actual income, was significantly associated with reduced job satisfaction across most measures except for ‘Freedom to choose your own method of working’ and ‘Amount of variety in your job’ (see Supplementary Figure S8). It was also significantly associated with increased intentions to continue medical work abroad or leave direct patient care within 5 years (see Supplementary Figure S9).

A focus on partner GPs showed there were similar findings to the main analysis, with a consistent relationship between higher actual income and most measures of job satisfaction (see Supplementary Figure S10), as well as a non-significant relationship between actual income and intentions to quit (see Supplementary Figure S11). Higher expected income, conditional on actual income, was significantly associated with reduced job satisfaction across all measures (see Supplementary Figure S12), and increased intentions to quit across all measures (see Supplementary Figure S13).

Stratifying by age group demonstrated that the relationship between actual income and job satisfaction was less consistent and non-significant for many income bands in younger age groups, specifically those aged <40 years (see Supplementary Figure S14) and those aged 40–50 years (see Supplementary Figure S15). In contrast, there was a consistent relationship between higher actual income and most measures of job satisfaction in GPs aged >50 years, except for ‘Physical working conditions’, ‘Your colleagues and fellow workers’, and ‘Amount of variety in your job’ (see Supplementary Figure S16). Similar to the main analysis, there was an inconsistent relationship between actual income and intentions to quit across all age groups (see Supplementary Figures S17–S19).

Higher expected income, conditional on actual income, was consistently associated with reduced job satisfaction across all age groups (see Supplementary Figures S20–S22). The relationship between expected income, conditional on actual income, and intentions to continue medical work abroad within the next 5 years was highest in GPs aged <40 years (see Supplementary Figures S23–S25). Although still significant, the smallest relationship between expected income, conditional on actual income, and intentions to quit were for the GPs aged >50 years.

## Discussion

### Summary

Data were analysed from four waves of the GPWLS between 2015 and 2021 to examine the relationship between job satisfaction, intentions to quit, and both actual and expected income. Higher actual income was significantly associated with higher job satisfaction across most domains, except for ‘Amount of variety in your job’, ‘Your colleagues and fellow workers’, and ‘Physical working conditions’; however, actual income showed no significant association with intentions to leave direct patient care or medical work entirely within 5 years. In contrast, a higher expected income, conditional on actual income, was significantly associated with lower satisfaction across all domains and significantly higher intentions to quit, including working abroad, leaving direct care, and leaving medical work entirely within 5 years. Stratified analyses indicated that the relationship between higher expected income, conditional on actual income, and job satisfaction was stronger among GP partners and older GPs.

### Strengths and limitations

Although previous studies have examined the relationship between actual income and GP job satisfaction, to the authors’ knowledge, no previous study has examined the relationship between expected income and job satisfaction for GPs in England. A further strength of the analysis is that the data were from the GPWLS, which has responders from GPs throughout England, covering four survey waves over 6 years. Multiple domains of job satisfaction and intentions to quit were assessed, providing a comprehensive set of outcome measures. Importantly, the authors drew on a direct measure of expected income, rather than assuming a comparator group or inferring expectations indirectly, which allowed for the capture of gaps between expected and actual income more accurately.

Limitations of the analysis include the fact that there may have been selection bias in the survey if GPs with lower job satisfaction were more willing to complete it because they saw it as an opportunity to encourage policy change. In addition, responders to the GPWLS may not have been fully representative of the GP population in England; survey weights were used to attempt to address this issue. Differences in survey methodology and contextual factors over time were also a limitation as job satisfaction was measured at different times of the year for each survey, and an online survey was used in 2021; indicators for each survey wave were included in the regression models to account for these differences. It is also important to consider that survivorship bias might be an issue when interpreting the findings; GPs who stated intentions to leave in earlier waves may have left NHS primary care by the time the later waves were conducted, which could have led to job satisfaction being higher in later waves.

### Comparison with existing literature

Similar to the findings of Allen *et al,* ^17^ higher actual income was found to be associated with greater job satisfaction for GPs. This finding also complements those of the Commonwealth Fund’s 2022 survey of primary care physicians, which reported that UK GPs were among the least satisfied with income and with practising medicine globally.^16^ This study’s finding that actual income was an inconsistent predictor of intentions to quit is also supported by the existing literature: Marchand and Peckham conducted a systematic review of qualitative and quantitative evidence on GP recruitment and retention, and emphasised how intrinsic and idiosyncratic factors were more important determinants than extrinsic factors, such as financial incentives.^7^

The findings related to expected income, conditional on actual income, and job satisfaction and intentions to quit align with the behavioural economics literature, which emphasises the role of relative income and expectation gaps in shaping job satisfaction.^22,26^

### Implications for research and practice

This study has important implications for future research and workforce strategy. The gap between expected and actual income appeared to be more consistently related to reduced job measure and intentions to quit, than actual income. This underscores the relevance of income expectations as a psychological driver of job satisfaction and retention. Future qualitative research could further investigate what factors shape income expectations among GPs as this could inform the development of interventions to manage them and improve retention. As an example, if comparisons with peers working in hospitals or other sectors are driving income expectations, policies could be introduced to ensure GP and hospital consultant reimbursement increases at the same rate or according to average economy-wide earnings, thus reducing GPs’ income being outstripped by their peers’ in a way that inflates expectations.

More broadly, improved transparency regarding what GPs earn for different job roles and hours worked may help to address dissatisfaction caused by any discrepancy between income expectations and actual income. It is notable that current statistics on GP incomes are not presented by numbers and time of hours or sessions worked;^27^ as such, it can be challenging for GPs to build accurate expectations of what they should earn. Finally, it is important to understand the intersections between actual income, income expectations, and working conditions. Alongside addressing income expectations to the extent possible within tight budgetary constraints, interventions to improve working conditions — such as reducing administrative burden, earmarked time for professional development, and improved triage systems to manage patient demand — may offer a more-comprehensive and sustainable strategy for improving GP recruitment and retention.

Prior research has found that GPs working in areas of higher deprivation earn less relative to GPs in areas of lower deprivation.^28^ This is worth considering when interpreting the results that suggest that GPs on lower incomes, along with GPs who have a higher gap between their expected and actual income, experience lower job satisfaction. If the higher expected income gap is somewhat driven by the level of deprivation of a GP’s practice, policies to reduce salary inequity between GPs working in areas with different levels of deprivation areas should be considered; this is especially important in the context of health inequalities in areas of deprivation, where GP retention in higher deprivation areas may be important.

## Data

In accordance with the terms of participant consent, the dataset analysed in this study is not publicly available.
